# Tools of the data detective: A review of statistical methods to detect data and result anomalies in psychology

**DOI:** 10.1177/09593543241311861

**Published:** 2025-02-01

**Authors:** Gabriel Crone, Christopher D. Green

**Affiliations:** York University; York University

**Keywords:** data fabrication, raw data, review, statistical tools, summary statistics

## Abstract

In psychology, it is largely assumed that researchers collect real data and analyze them honestly—that is, it is assumed that data fabrication seldom occurs. While data fabrication is a rare phenomenon, estimates suggest that it occurs frequently enough to be a concern. To this end, statistical tools have been created to detect and deter data fabrication. Often, these tools either assess raw data, or assess summary statistical information. However, very few studies have attempted to review these tools, and of those that have, certain tools were excluded. The purpose of the present study was to review a collection of existing statistical tools to detect data fabrication, assess their strengths and limitations, and consider their place in psychological practice. The major strengths of the tools included their comprehensiveness and rigor, while their limitations were in their stringent criteria to run and in that they were impractical to implement.

In psychology, there is a widely held assumption that research is done in an honest way. That is, it is largely assumed that psychologists conduct their research by collecting real data and presenting statistical results that accurately reflect said data. This assumption is fundamental to scientific progress in psychology, because it enables scientists to build upon one another’s work, and ensures the public benefits from scientific discoveries. Despite the importance of said assumption, however, it has not always held true. In recent decades, there have been many cases in which researchers within psychology (and other sciences) have committed research misconduct.

Research misconduct presents itself in three main forms: plagiarism (falsely presenting someone else’s thoughts as one’s own), data falsification (the intentional alteration of existing real data), and data fabrication (intentional attempts to create new, false data), which together are known as falsification, fabrication, and plagiarism (FFP; Steneck, 2006). Each form is concerning in its own way, but for the purposes of the present review, we will focus on data fabrication.

Some cases of data fabrication are particularly well-known for how extreme they are. For example, the Retraction Watch Database (Centre for Scientific Integrity, 2024) updates a list of individuals with the highest numbers of retracted papers. Within the list of 31 researchers (as of April, 2024), there are a total of 1716 retracted studies (for a per-individual average of 55 retracted research papers).

Beyond specific cases, there is a research literature that aims to establish the prevalence of scientific misconduct in psychology. In a recent systematic review, Stricker and Günther (2019) analyzed a multitude of journal articles from PsycINFO. They found that 0.82 per 10,000 (0.008%) articles were retracted due to research misconduct (e.g., data fabrication). In another systematic review, Fanelli (2009) reviewed 21 surveys in which scientific researchers were asked to anonymously disclose if they, or other researchers they knew, fabricated data. Across all studies, approximately 1.97% of scientists self-admitted to having participated in research misconduct themselves (at least once), and 14.7% of scientists admitted to having observed a colleague committing it.

Less formally, one can approximate the extent of misconduct by considering the prevalence of retracted studies due to data fabrication. The blog *Retraction Watch* (https://retractionwatch.com) has a large database of retractions from a variety of journals across many academic disciplines, including psychology. As of the time of writing this paper (April, 2024), their database (with more than 47,000 retractions total) contains at least 1630 retractions due to data being fabricated (Centre for Scientific Integrity, 2024). Given these different pieces of evidence, it is rather clear that while a relatively rare phenomenon, data fabrication is an issue of concern in psychology and other sciences.

Data fabrication is harmful to psychology and science for several reasons. First, it is detrimental to the credibility and trustworthiness of science. Studies based on fabricated data are unable to provide support for their claims. (The claims might be true, but there is insufficient reason to believe them to be true.) Thus, such studies muddle the trustworthiness of claims made in the research literature. Moreover, the mess they create is rather difficult to clean up: well after studies get retracted, they tend to linger in the form of postretraction citations. These citations occur when new articles cite retracted papers and are problematic because they provide the illusion of empirical support. The problem is ongoing, and it is especially prevalent within many sciences, including medicine (see, e.g., Budd et al., 2016; Hsiao & Schneider, 2021) and psychology (see, e.g., Yang et al., 2024). Data fabrication also damages the public’s perception of science. Since many instances of research fraud are heavily publicized, it sows the seeds of scientific mistrust, which is a volatile and damaging belief for the public to hold.

Additionally, data fabrication harms those around the fabricator, including the fabricator’s students, collaborators, and the public. Students often face backlash for being associated with their supervisor (even if they had nothing to do with the fabrication)^1^ and collaborators lose time, money, and funds spent working with fabricators. Lastly, and more practically, it wastes taxpayer money that should be going toward high-quality research.

Given such concerns, what can be done to deter and detect data fabrication? There exist several tools to help achieve this goal. However, before discussing these tools, it is important to first highlight some well-known instances of data fabrication.

## (In)Famous cases of data fabrication

### Diederik Stapel

Perhaps the most well-known case of data fabrication in psychology was that of the Dutch social psychologist Diederik Stapel. Stapel was once praised as a “superstar” within social psychology, well-known for producing novel social psychological studies that captured the attention of the scientific community and the public. Stapel would always “collect” data for his students and collaborators without any supervision. When it was noticed that Stapel’s published research results always seemed to be too perfect, it raised many red flags. Eventually, three anonymous young researchers began noticing anomalies (e.g., large numbers of duplicated rows) in Stapel’s data sets, prompting them to report Stapel to the department head of Tilburg University. Soon, the three universities Stapel had previously worked for initiated an investigation (see Levelt Committee et al., 2012). It revealed that Stapel had fabricated entire data sets for studies or altered existing data sets in a manner where statistically significant (and moderately sized) effects were (nearly) always acquired.

After it was finalized, the written report described the extent of Stapel’s massive fraud: he had fabricated data for 55 of his own publications and 10 students’ Ph.D. theses. The extent of the data fraud was staggering, and it had a lasting impact within the psychological research community (for a review, see Bhattacharjee, 2013).

### Dirk Smeesters and Lawrence Sanna

Two other cases of importance are those of Lawrence Sanna and Dirk Smeesters. Sanna was a psychologist known for his work in decision-making and morality. Similarly to Stapel, his results also seemed too good to be true, which grabbed the attention of Uri Simonsohn. Simonsohn is a professor of behavioural science who, in addition to his professorial work, investigates anomalous data within published research reports. When examining Sanna’s results, he found that they contained anomalies such as incredibly strong effects (relative to similar studies) and abnormally small standard deviations (SDs) related to demographic estimates (for details, see Simonsohn, 2013). Simonsohn reached out to Sanna’s co-authors, who would relay additional concerns to the University of North Carolina at Chapel Hill, Sanna’s previous employer. Subsequently, Sanna left his current institution, and had five of his papers retracted, indicating likely responsibility for what occurred (for a full review, see Yong, 2012).

Smeesters was a social psychologist working at Erasmus University who did research on priming effects. In one of his studies, Simonsohn noticed that the results were too closely aligned with the predicted hypotheses, prompting a fuller investigation. Using simulations, Simonsohn found that the data were highly unlikely to have occurred via a natural sampling process. Simonsohn communicated with Smeesters, before a research ethics board investigated his work, resulting in his resignation. Smeesters admitted that he omitted data to achieve significance (i.e., *p*-hacked); however, he attempted to minimize his actions by insisting that such practices—“data massaging,” in his words—were already common in psychology. When the committee wanted to verify if the data were fabricated rather than just *p*-hacked, Smeesters reported that the data no longer existed due to the death of a home computer (for a full review, see Enserink, 2012).

The Smeesters and Sanna cases both highlight the utility of using purely statistical methods to detect data fabrication. Simonsohn (2013) details how he managed to detect the abnormalities in Sanna’s and Smeesters’ studies using various statistical methods (which are to be discussed later).

### Nicolas Guéguen

Another case worthy of mention is that of Nicolas Guéguen, a French social psychologist. Guéguen was known for conducting studies that tested old-fashioned social stereotypes related to sexual attraction. In one paper, for example, he claimed that men were more likely to help women wearing high heels rather than women wearing flat shoes. His studies were eventually investigated by two data sleuths: Nick Brown and James Heathers. (For some context, the two researchers regularly investigated many instances of data anomalies within published research papers.) They wrote a comprehensive 50-page report on the wide variety of suspicious anomalies in Guéguen’s work—from statistical errors to methodological implausibilities.^2^ With the sizable quantity of evidence, Brown and Heathers reported Guéguen to authorities from his university. The result was slightly underwhelming: of the 10 studies Brown and Heathers investigated, the university only called for the retraction of two of his papers. Only one has since been retracted (see O’Grady, 2019).

This case is noteworthy for two reasons. First, it emphasizes the importance of having whistleblowers report fraud when it is noticed. And second, it demonstrates that it often takes a long time to detect and properly handle research misconduct—if at all. From the time it was originally reported, of the 10 articles of Guéguen’s that Brown and Heathers (2017a) noticed, it took four years to have only a single paper retracted.

### What can be learned from cases of data fabrication?

The cases reviewed highlight three important aspects of data fraud that are worth discussing. The first aspect involves how difficult fraud is to detect and report. Scientists—especially those whose research is respected by many professionals, organizations, and the public—tend to be highly trusted, and when someone is highly trusted, they are rarely suspected of wrongdoing.^3^ For example, many social psychologists held Stapel, and his research, in high regard, so many did not initially question Stapel’s unusual practice of collecting data for his students. Additionally, since fraud is such a serious accusation, there is a lot of risk in coming forward with accusations.

The second aspect of fraud has to do with the general lack of preventative measures in place. In all the cases outlined, the fraud was only caught when the individual(s) took notice of anomalous results or data. If said individuals had not taken it upon themselves to uncover evidence on their own, and take the risk in accusing them, the fraud might never have been caught. Despite calls toward more open-scientific practices (e.g., open-data & code), research data and analyses are also infrequently made available (Hardwicke et al., 2022), making it more difficult to investigate potential fraud.

The third aspect of fraud has to do with why individuals partake in it. Within the discussed cases, when one partook in fraud, they often did so for professional gain. For example, by publishing novel, “exciting” results, they accrued more publications, and with them more professional success, government funding, and high acclaim. This bias toward “novel/exciting” results, unfortunately, is precisely how the very system of scientific publishing currently operates. For example, before he fabricated data, Stapel struggled to make a name for himself; after he began fabricating data, he quickly rose up the professional ranks (Bhattacharjee, 2013). Many of Sanna’s and Smeester’s (now-retracted) papers were found in high-impact journals (e.g., *Cognition*, *Nature*), which certainly benefited their careers.

In summary, it is important to discuss cases like these because they not only serve as important indicators of the dangers and harms that data fabrication can cause, but they also served as the motivation to create statistical methods to detect data fabrication.

## Data fabrication overview—Terms and methods

Before proceeding, it is important to clarify what is meant by “data fabrication.” At present, *data fabrication* entails any intentional attempt(s) to create new, false data, to produce some desired outcome (e.g., statistically significant results or large effects). This definition should not be confused with less serious (yet equally important) questionable research practices, such as *p*-hacking. *p*-hacking occurs when researchers use “researcher degrees of freedom” (flexibility in analysis decisions) to produce a significant statistical result when none would otherwise exist (see Simmons et al., 2011). Data fabrication, in contrast, involves a more explicit and purposeful act, in which false data is generated, which is not a permissible researcher degree of freedom.

Because fabricated data is so harmful to psychology and other sciences, finding ways of detecting it would help ensure that its prevalence decreases. As some scholars have pointed out (e.g., Hartgerink et al., 2016; Simonsohn, 2013), detection is key in deterring data fabrication because the more awareness they have that an undesirable behaviour can be detected, the less incentive fabricators have. To help detect data fraud, researchers have developed several statistical tools.

There are two main types of statistical tools. The first are tools that specifically examine raw data (hereafter, *raw data tools*), and the second are those that examine summary statistics (hereafter, *summary statistics tools*; see Hartgerink et al., 2016). Each method has its own underlying statistical basis, strengths and limitations, and history of use.

Aside from Hartgerink et al.’s (2016) study, research has often only considered each method independently. Moreover, within Hartgerink et al.’s (2016) research, certain newer methods were not discussed (e.g., the GRIM, GRIMMER, and SPRITE; maximal positive controls).

## Current research

The main goal of the current review is to synthesize the literature regarding various popular data fabrication detection methods in psychology. Moreover, the purpose is to acquire a more complete understanding of: (a) how each method works on a basic statistical level, (b) the (major) applied usages of each method, (c) each method’s strengths and limitations, and (d) the existing software to run methods.

More broadly, the current research will also consider the potential applicability of the methods in detecting data fabrication within published research, with a call to use the methods as carefully, critically, and conscientiously as possible.

The present research extends Hartgerink et al.’s (2016) review in that it describes additional methods that were not discussed (e.g., GRIM. GRIMMER, etc.), explains methods in a more accessible way, and provides further information regarding software implementations for the various methods discussed, which can be found in the online supplemental materials.

## Literature review

### General note of caution

Before describing the various methods in depth, it is important to discuss some notes of caution. First, as Hartgerink et al. (2016) stress, many of the methods originated once data fabrication was already suspected. Often, when detecting data fabrication, there was an indicator that something within the research was amiss. The effect sizes were too large; the analyses and data were too clean; the results were too perfect. It was only through having such indicators that individuals were able to use statistical methods to objectively evaluate their suspicions. Given the case-by-case nature by which methods are developed and tested, it is possible that they may not generalize well to other (potentially undetected) cases of data fabrication.

Additionally, the methods discussed are not to be considered the panacea to “cure” the ills incurred by data fabrication in psychology. Each method has its own limitations, which govern the extent to which they can be applied to suspected cases. As such, much like many existing statistical inferential tests in psychology, the methods are buttressed by assumptions that, if violated, render the results uninterpretable and potentially misleading. Lastly, if the methods support the idea that data have anomalies, this alone is insufficient to conclude that misconduct occurred; rather, it only indicates that there *might* exist misconduct. Since accusing individuals of fraud is risky for all parties involved, the “burden of proof” is usually set much higher for accusers. Thus, even in cases where researchers believe there to be a “smoking gun,” fraud should never be alleged unless there is incontrovertible evidence.

### Raw data tools

#### Newcomb-Benford law

The first raw data tool is the Newcomb-Benford law (hereafter NBL), a mathematical law that describes the expected frequencies of leading digits (i.e., natural numbers from 1–9) within random, ratio-level continuous data. It was first discovered by Newcomb (1881), who noticed that in printed books of logarithmic tables, “the first significant figure is oftener [sic] 1 than any other digit, and the frequency diminishes up to 9” (p. 39). Newcomb formalized this observation mathematically by defining an expression for the expected frequencies of first and second digits. However, he lacked empirical data to support his claim. Benford (1938), 57 years later, collected such data and largely popularized the law (Berger & Hill, 2011).

The phenomenon the law describes seems rather unintuitive at first. When asked what the expected frequency of any given leading digit is, one might expect that each digit has an equal probability (with each digit having a 10% chance); that is, the distribution of expected digits is uniform. However, due to the nature with which leading digits appear in raw, continuous data, the distribution of expected digits, from 1–9, is a function of a logarithm:

$$F_{a}=log_{10}\left(\frac{a+1}{a}\right)$$

(Benford, 1938, p. 554), where *F_a_* is the frequency of a leading digit *a*, and *a* is a natural number from 1 to 9.

From this law, a distribution of expected values for leading digits 1–9 can be derived. Indeed, this law was confirmed by Benford (1938) himself. After he collected a large data set consisting of 20,229 data values from across 22 diverse, real-life domains (e.g., data on the length of rivers, size of populations, death rates), he observed that the NBL fit the data well. Subsequent articles have provided other empirical data to support the law (for a review of such articles, see Raimi, 1976).

Since the NBL describes expected frequencies for leading digits within data, deviations from the NBL may indicate that something within the data is amiss. To test this, one could easily run a statistical test (e.g., χ^2^ goodness-of-fit test, *z*-test) to compare if the expected distribution of leading digit frequencies matches the distribution predicted by NBL. This is precisely how the NBL is often used in domains such as fraud detection (Nigrini, 2012).

Could the NBL be applied to data fabrication? Earlier research (e.g., Hsü, 1948; Kubovy, 1977) suggested not. When human participants were asked to generate meaningless random numbers, their data were undetectable by the NBL. However, these studies were criticized because the tasks participants completed were not generalizable to real-life situations (Diekmann, 2007). For example, when one fabricates data, they do not merely generate completely random, arbitrary numbers; they generate numbers with meaning. When asked to generate numbers that had more meaning (e.g., regression coefficients), participants generated fabricated data that were detectable by the NBL (Diekmann, 2007).

Subsequent studies have used the NBL to differentiate fabricated data (from retracted studies) from genuine data. Such research has done so successfully within the fields of medicine (Hüllemann et al., 2017), economics (Tödter, 2009), accounting (Horton et al., 2020), and biology (Eckhartt & Ruxton, 2023).

Despite the NBL’s wide applications, it has many requirements that limit its usefulness in psychology: it is restricted to continuous, ratio-level data, which ideally must range from 1 to 100,000 and must not be overly rounded or truncated (Hartgerink et al., 2016); it is not scale invariant (Berger & Hill, 2011); it cannot be applied to data that are normally, uniformly, or exponentially distributed (Berger & Hill, 2011; Eckhartt & Ruxton, 2023), and it requires at least 250 observations for adequate statistical power (Joenssen, 2014).^4^ Given these limitations, the NBL would have a low level of applicability in psychology research, because its stringent criteria are rarely met.

#### Multivariate associations

Hartgerink et al. (2016) pioneered the method of Multivariate Associations. As they explain, in each research literature, there exist specific associations (e.g., correlations) between variables that frequently occur. For example, one might expect that in a sample of studies examining depression and anxiety, the measures of depression and anxiety are highly correlated. One could roughly gauge such associations by finding several papers related to an effect and computing the correlation between the relevant variables. With enough correlations, one could create a distribution of “expected” correlations and compare the suspected study’s correlation(s) (between specific variables) to the expected distribution. The technique is useful because fabricators might miss, or simply have difficulty emulating, multivariate associations in their own (fake) data (Hartgerink et al., 2016).

In one of their own studies (Study 2), Hartgerink et al. (2016) asked participants to fabricate a plausible raw data set for a (fictitious) classic Stroop experiment, and undertook a multivariate analysis. To do so, they first gathered data from the Many Labs 3 Project (one of several large-scale psychology replicability projects; Ebersole et al., 2016), computed correlations for four variable pairs from the data, created a parametric distribution based on these correlations, computed the same correlations within each fabricated data set, and ran statistical tests to determine if the computed correlations from the fabricated data sets were unlikely given the parametric distributions. They found that using multivariate associations was a rather useful method to differentiate fabricated from nonfabricated data. For example, when plotting the distribution of the fabricated and genuine data, the distribution of the fabricated data correlations had far more variability relative to the genuine data.

Beyond the Hartgerink et al. (2016) study, no other primary uses of multivariate association analysis in detecting data fabrication could be found in the research literature. More research is needed to determine if the method is a reliable indicator of fabricated data.

The method is more flexible since it lacks the NBL’s strict criteria. However, its largest limitation is that raw data must be available for several studies on a specific effect (Hartgerink et al., 2016). Said data sets might not exist if the phenomenon under study is relatively novel. Another possibility is that if comparable studies exist, it might be difficult to acquire their raw data (since open data sets are relatively uncommon; Hardwicke et al., 2022).

### Summary statistics tools

#### Variance analysis

In some previous cases of data fabrication (e.g., in the Smeesters and Sanna cases), one particularly obvious anomaly within reported statistical data had to do with the similarity of reported statistics. When statistics are reported, it is indeed quite rare to find descriptive statistical information that contains duplicates (e.g., identical SDs) within the same study or research article. In fact, observations with a high degree of similarity might indicate something amiss. This exact insight was the inspiration for a technique pioneered by Simonsohn (2013) entitled variance analysis. Variance analysis involves examining the variances of the variances (called the dispersion of the variance; Hartgerink et al., 2016) to determine the statistical likelihood that said variances occurred assuming a random sampling procedure was done in collecting the data. Obviously, the more identical (or very similar) SDs are to one another, the more anomalous the reported descriptive statistics are.

Hartgerink et al. (2016) explain how one would go about quantifying the extent to which the dispersions of the variances would be theoretically computed. In essence, the technique to quantify the probability that a particular set of variances are unusually similar would involve two steps: (a) compute a theoretical distribution of standardized variances and (b) perform bootstrapping with said distribution to deduce what the expected distribution of the dispersion of variances would look like. Details of performing said procedure can be found within Hartgerink et al. (2016, pp. 6–8).

Once the final expected bootstrapped distribution is arrived at, one can test how “extreme” the dispersion of variances observed in a study is (usually reported as the number of simulations that yielded the same or a more extreme similarity of variances, relative to the number of total simulations performed). Similarly to multivariate analyses, one could also run a statistical test to determine if the acquired dispersion of variance is statistically different from the rest of the bootstrapped distribution. Often, the test is most useful when performing a comparison between “control” studies assumed to be based on genuine data and a target study under suspicion.

Simonsohn (2013) originally used this technique to determine that the results reported in a Sanna paper and a Smeesters paper were highly implausible. Specifically, Simonsohn examined a Sanna paper claiming that higher elevations “caused” people to act more prosocially. In a summary table across all three studies within the paper, Simonsohn noticed an anomaly: the reported means were quite different, but the SDs were almost identical. By running a variance analysis on the SDs, he found that the SDs that Sanna had acquired were very unlikely (only occurring 1.3% of the time). Concerned that the scaling was too different across studies, Simonsohn divided the dispersion of the variances by the standard error (SE) of the pooled SDs, resulting in what he calls a Ψ (“psi”) estimate. Using said technique, he found that the original study’s SDs showed a Ψ of 0.174. Assuming a random sampling procedure, this value was statistically absurd: the value should, theoretically, be near zero, but relative to all other 100,000 simulations, such a rare Ψ value would have only occurred 0.015% of the time.

Simonsohn also conducted a similar procedure with a Smeesters study, specifically with a table of reported means and SDs. Using variance analysis, he computed a Ψ of 0.308 for the study; again, the value was exceedingly unlikely within the 100,000 simulations (occurring only 0.021% of the time). These examples show the utility of using variance analysis to detect whether dispersions of variances are exceedingly unlikely assuming a random sampling procedure was done.

Beyond the studies outlined presently, there do not exist further applications of said technique in the literature.

#### Extreme effect sizes

Another technique to detect anomalous results within the literature—described by Hartgerink et al. (2016)—involves considering the effect size a particular study reports. An effect size (or ES) is the difference between or among a group of variables (Flora, 2020). Effect sizes can be either unstandardized (e.g., mean group differences) or standardized (where it is in the metric of SDs; e.g., Cohen’s d [Cohen, 1988], R^2^ statistics). For the purposes of examining extreme effect sizes, standardized effect sizes are frequently used due to the ease with which they can be compared across studies (Hartgerink et al., 2016).

The method itself is rather simple: when the reported effect sizes tend to be substantially larger than those that are often reported within an area of investigation, the studies themselves are considered anomalous. This fact has been demonstrated empirically with correlation coefficients (Akhtar-Danesh & Dehghan-Kooshkghazi, 2003). This also makes logical sense, too; the more abnormally large an effect size is relative to comparable literature, the more anomalous.

How can researchers quantify just how extreme an effect size is? They can do so by comparing the effect size to those typically found in comparable literature. To achieve this, researchers first survey comparable literature to determine a typically sized effect, then create a hypothetical distribution of similar effects, and compare the effect sizes within the target study to see if they deviate significantly from said distribution of typical effects (Hartgerink et al., 2016).

Suppose, for example, that a depression researcher fabricates a large standardized mean difference of *d* = 0.95 comparing an experimental group who received an antidepression drug to a control group that did not in two studies. Further suppose that a review of similar depression treatments yielded the following *d* values: 0.65, 0.25, 0.32, 0.76, 0.32, and 0.21. One could then run a Wilcoxon signed rank test (more robust version of a single sample *t*-test) using *d* = 0.95 as the mean; the test shows that *d* = 0.95 is statistically different from the other *d* values.

In the context of extreme effect sizes, standardization is often a sticky issue because if one standardizes two measures to the same metric, they may still not be directly comparable. Thus, effects for studies are often compared only if they share a similar metric. As with the previous methods involving examining comparable literature, it is limited because the comparable research must have already been done.

##### Maximal positive controls

While not a statistical test in itself, a fascinating, relevant submethod used to test for extreme effects sizes is called a maximal positive control (Hilgard, 2021). In experimental designs, there are two types of control: positive and negative. A *negative control* occurs when a group receives none of a given treatment (e.g., wait-list control, placebo group) so that this group can be compared to the one receiving a treatment. In contrast, a *positive control* occurs when a group receives sufficient or abundant treatment, usually with a known outcome, to compare to other groups that do not. In a *maximal positive control* study, researchers design an experiment with the express purpose of acquiring the highest theoretical plausible effect sizes (Hilgard, 2021). Such effects act as a “ceiling” for similar experimental research; if an effect size exceeds the ceiling, it indicates that the effect size in question is too large and thus anomalous.

Hilgard (2021) used this technique in a few case studies, in which he found that several studies within the literature often tended to exceed the maxima he had arrived at through executed maximal positive controls, suggesting that these studies contained abnormally large effect sizes. The method is still in its infancy and little attention has been paid to it. Only one instance of the technique being applied was found in the literature. Zaini (2022) used this technique to determine if the Macbeth effect, originally discovered by Francesca Gino—an ex-Harvard Business School professor accused of falsifying data—was real.^5^ Zaini found that Gino’s original study’s effect size exceeded the ceiling from her maximal positive control, discrediting Gino’s finding.

#### *P* value analysis

An additional summary tool is known as the *p* value analysis method, pioneered by Hartgerink et al. (2016). For any given study, the *p* value distribution is expected to be either positively skewed (if there is, hypothetically, support for a detected effect) or uniform (if there is hypothetically no support for an effect; Fisher, 1925). Thus, distributions that deviate from those expected shapes (e.g., a negatively skewed distribution) would be considered anomalous. The way that Hartgerink et al. (2016) go about investigating whether the distributions deviate is through a mathematical modification of Fisher’s method (Fisher, 1925). The original Fisher method has been used as a meta-analytic tool, which could detect that a distribution of *p* values is positively skewed (indicating support in favour of said effect). The modified version of the test, Hartgerink et al. (2016) argue, allows one to test against the same null as Fisher’s test, but under the alternative that the “results are closer to their expected values than expected under the null” (p. 6). In other words, the new test allows one to determine if the obtained *p* values are abnormally similar. For example, one could take a sample of *p* values from a study and input it into a Reversed-Fisher test. Suppose a researcher collected three nonsignificant *p* values in a study: $p_{1}=0.918,\;\;p_{2}=0.920,p_{3}=0.911.$ When imputed into the Reversed-Fisher test (via the ddfab R package; Hartgerink, 2024), the result is statistically significant, F(6)=14.89,p=.021, indicating that the *p* values were excessively similar. The method’s authors used it to differentiate fabricated from genuine data, finding that the overall *p* value for the test was significant when data were fabricated, but nonsignificant when data were not.^6^

An issue of this method is that if tests do not specify whether they are 1-tailed or 2-tailed, it could result in *p* values that are excessively large, contributing to potential erroneous interpretations (Hartgerink et al., 2016). As well, if a study’s reported statistical tests are more complex, or are not constant across *p* values, then simply plugging *p* values into the Reversed-Fisher method would produce inflated estimates.

Additionally, there are reasons beyond data fabrication that could explain failing Fisher’s test. For instance, if there is rather intense *p* hacking occurring within one’s research study (though, technically, no data fabrication), the resulting *p* value distribution would be negatively skewed (Simonsohn et al., 2014).

#### GRIM, GRIMMER, and SPRITE

While not inherently intended to detect data fabrication, it is worth describing three techniques that examine the accuracy of reported summary statistics within research papers: the GRIM, GRIMMER, and SPRITE techniques.

The GRIM (the granularity-related inconsistency of means) technique (Brown & Heathers, 2017b) is a mathematical method used to tell if reported means within survey studies are mathematically plausible. Given a specific number of survey responses and a given sample size, there exists a selection of means that are mathematically possible. If the reported mean is *not* one of said possible means, then an error in the reporting of the mean occurred, and said mean is said to be *inconsistent*.

To illustrate this, imagine that a researcher reported the following: “We asked the control group (*N* = 25) to report their mood on a 1–7 scale (1 = very happy, 7 = very unhappy). They had a very high negative mood (*M* = 6.98).” The sum of the scores must range from 25 (if everyone responds with 1) to 175 (if everyone responds with 7), and the sample size is constant (25), so a mean can be computed for all values from 25 to 175. The reported mean of 6.98 is not within the set of possible means (the closest means are: 174/25 = 6.96 and 175/25 = 7.00), so it fails the GRIM test and is thus inconsistent.

An inconsistent mean likely represents carelessness on behalf of the researcher, but it may (in rare cases) represent an instance of dishonest reporting or fabrication. In any case, Brown and Heathers (2017b) suggest that such an inconsistency should be detected and reported back to the original researchers for correction. The test itself is based on a mathematical generalization that computes the plausible means, making it rather efficient.

Brown and Heathers (2017b) have used their own method on a sample of articles from several psychology journals. Using the GRIM test, they found that around half of the articles examined contained at least one inconsistent mean, and 20% contained more than one inconsistent mean. With their data, they contacted the authors for nine papers, and corrections for the impossible means were made.

A limitation with the GRIM method is how, with larger samples, the possible means become more similar, and so with rounding considerations, the test is much less likely to detect an inconsistency. Specifically, as a rule, Brown and Heathers (2017b) suggest that the technique does not work in cases where the per-cell sample size is greater than 100.

The GRIMMER technique (Granularity-Related Inconsistency of Means Mapped to Error Repeats; Anaya, 2016)^7^ tests whether reported SDs are mathematically plausible both on their own and with respect to their reported means. To propose the test, Anaya (2016) arrived at two clever observations. The first observation was that with a given sample size (so long as *N* ⩾ 5), the plausible computed variances follow expected patterns. When computing variances with a sample size of *N* = 5, Anaya noticed that the fractional parts of the variances repeated themselves. This exact repeating pattern, however, was different when the integer parts of each variance were even (called the Even Pattern, or EP) than when they were odd (called the Odd Pattern, or OP). Only when the sample size was even, did the EP = OP. The second clever observation was that with reported means, only a limited number of possible variances could be computed from them.

Making use of these observations, Anaya (2016) formalized the GRIMMER test as a composite test: the first runs a simple GRIM test on the mean, the second checks if the reported variance (converted from the SD) follows an EP or OP, and the third checks if the reported variance is possible given the reported mean and sample size. If any test fails, the mean/SD combination fails GRIMMER. When this occurs, it may indicate that the reported values were due to an honest error or sloppiness, often warranting a correction. In some cases, however, it might suggest that the underlying data were fabricated, in which case, Anaya (2016) suggests that GRIM tests alone are usually enough to detect fraud. Moreover, Anaya (2016) heavily cautions, “With great power comes great responsibility . . . I don’t intend for the test to be used to conduct witch hunts” (p. 10).

The technique is limited insofar as it does not reliably work with very small samples (if *N* < 5), nor does it work well if the sample size is too large (since, just as with GRIM, when the granularity becomes too fine, it is not possible to detect inconsistencies within a certain level of rounding). Additionally, the test often has very long computing times, and it assumes that negative values are possible within the data, so that even SDs that are mathematically impossible upon visual inspection might still technically pass the GRIMMER test.

The final of the trio of related techniques is the SPRITE method (Sample Parameter Reconstruction via Iterative TEchniques; Heathers et al., 2018).^8^ SPRITE takes any given sample statistics and simulates data that align with a set of desired inputted parameters (e.g., SD, skew, kurtosis). Under the hood, the SPRITE algorithm starts by simulating a data set that produces the inputted mean, but not necessarily other parameters. Then, it checks if the other parameters are met, and if not, adjusts the simulated data again and again until the data produce the desired parameters. Once a final data set is arrived at, a distribution is plotted and numerically described. The idea is that by inspecting this output, one could determine if the summary statistical information from a study, when inputted into SPRITE, yields a realistic distribution. If, for instance, the distributions deviate from what one would reasonably expect to occur with real data, it would be anomalous.

For example, suppose that a researcher collects data from 1,000 participants and asks them on a 1–7 Likert scale to report their current hopes for the future. One would expect with such a high sample size that each response would be selected at least once. Thus, if one inputs the parameters from the study (e.g., the mean and SD of the responses) into SPRITE and observes that only two out of the seven responses must have been selected, then the reported statistics are anomalous.

The benefit of SPRITE is in how flexible and customizable it is. If a user, for example, wanted to add restrictions to the simulations, such as the produced data needing to have a specific skew, kurtosis, or relative frequency of scores, amongst many other specifications, it is quite easy to add in. Thus, the method is versatile as to what it can investigate.

SPRITE also has some disadvantages. The authors argue that using SPRITE in isolation is ill-advised. Other methods such as GRIM, GRIMMER, or simple visual inspection should accompany it. Additionally, SPRITE uses an algorithm based on a heuristic solution, rather than an analytic solution, so it is possible that the distribution SPRITE generates does not accurately reflect the real distribution underlying the data (Heathers et al., 2018). The authors also warn that SPRITE does not consider a range of technically plausible values; the result is but one potential “solution” that matches the reported mean and SD (and whatever other information is provided). Lastly, the developers also caution that SPRITE must be considered holistically and critically. That is, one should avoid applying general heuristics (e.g., expecting the generated distributions to always be normal, or only focusing on descriptive statistics of the generated distributions) and should instead consider several pieces of evidence presented before arriving at any claims.

In the context of data fabrication, the GRIM, GRIMMER, and SPRITE techniques have been used sparingly; however, there was one major case where all three methods were successfully applied to help detect studies with erroneously reported statistics. The case had to do with several studies published by the Cornell Food and Brand Lab. Anaya et al. (2017) investigated four papers published by the lab, where authors used the GRIM and GRIMMER methods (amongst other checks). In all, they found that there existed at least 150 inconsistencies and impossibilities.^9^ The SPRITE method was also applied by its creators on similar data from the Cornell Food and Brand Lab. In one study Heathers et al. (2018) investigated, they noticed a particularly strange mean/SD pair in describing the number of carrots that a group of 3–5-year-old children consumed. When SPRITE was run, it suggested that some children must have eaten 46 or more carrots! Considering the uses of GRIM, GRIMMER, and SPRITE (as well as some investigations by others), a total of 15 of the lab’s studies were retracted (for details, see Resnick & Belluz, 2018).

## Discussion

The purpose of this paper was to review various statistical methods that have been used to detect anomalies within published psychological literature. To that end, a diverse variety of methods were reviewed, each falling into one of two categories: (a) those that examined the raw data within a given study and (b) those that examined the reported summary and inferential information within the paper itself. The first type of method included the Newcomb-Benford Law (NBL) and multivariate associations, while the second type of method included variance analyses, extreme effect size analysis (and maximal positive controls), *p* value analysis, and various data consistency detection techniques (i.e., GRIM, GRIMMER, and SPRITE).

### Strengths and limitations of tools

The methods, collectively, have strengths. One is in their diversity. Each method works by targeting a unique aspect of a suspected study: its effect sizes, its *p* values, its raw data, or its reported descriptive statistics. In doing so, the methods could theoretically^10^ provide a holistic view of a research paper and have an improved ability to detect true cases of fraud. Additionally, since no case of fraud is identical to another (though many had similarities), having tools that target several aspects of a study (rather than focusing on only one or two) could be used to detect more cases than individual tools alone.

Another strength is the fact that methods frequently use more conservative criteria than typical hypothesis tests. Within the realm of data anomalies, having false positives is disastrous (as will be discussed shortly). Thus, many tools (especially Simonsohn’s [2013] variance analysis) use highly liberal criteria (i.e., more strict threshold cutoffs) to determine if data or results were anomalous in a particular regard.^11^ In other words, the “benefit of the doubt” is very high, with data fraud only being determined if the results were extremely statistically unlikely.

However, there were several limitations underlying the methods. First, despite some advantages, each method had caveats that posed considerable challenges in successfully implementing them. The NBL, for example, required rather stringent conditions to be applied, conditions that would seldom be met in psychological research. Many other methods could not be applied without having to carefully review literature that is comparable to the study in question, which is often impossible for research that is novel or uses novel methods.

In all, then, the methods are quite challenging to incorporate in practice. To illustrate this, Table 1 summarizes the various tests’ requirements. As can be seen, all the tools required at least accessible raw data, specific data or sample sizes, a review of comparable studies, or simulation methods, with some tools requiring two of these.

**Table 1.** Requirements to Run Each Data Fabrication Detection Tool.

Tool	Requirement			
	Accessible Raw Data	Specific Data or Sample Size*	Review of comparable studies	Simulation
NBL	✓	✓		
Multivariate Associations	✓		✓	
Variance Analysis				✓
Extreme ES			✓	
*p*-Value Analysis		✓		
GRIM		✓		
GRIMMER		✓		
SPRITE			✓	✓

*Note*. Check marks (✓) indicate that the requirement is needed for the tool. *: For NBL, the raw data must be ratio level and range from 1 to 10^6^; for *p*-value analysis, the tests must be correctly specified as 1- or 2-tailed; and for GRIM and GRIMMER, the reported sample size cannot be too large.

Additionally, except for the NBL, most methods were heavily under-researched. This might be due to the perception that data anomalies are an exceptionally rare occurrence, and so researching statistical methods to detect them would not be worthwhile. As established earlier, this idea is false, since current formal and informal estimates indicate that data fabrication occurs more frequently than is often believed. Alternatively, the dearth of research could be due to the case-by-case basis in which various methods are arrived at. Since most methods are created to investigate specific cases of data fabrication with its own suspected anomalies, other potentially anomalous studies are often not considered. Another explanation might be that many methods are based upon older existing statistical techniques, (e.g., bootstrapping, inferential statistical [primarily NHST] tests, and Fisher’s test), so checking the quality of these tests is not seen as necessary. I would argue that many of the methods are different enough from their more “classical” counterparts that they deserve more serious investigation. For instance, *p* value analysis involved a modified version of Fisher’s test, known as the reverse Fisher method (Hartgerink et al., 2016). For the former, research exists examining its valid uses and potential alternatives (e.g., Rice, 1990; Whitlock, 2005); for the latter, no such research exists. A similar pattern occurs for most of the given methods.

### Considering applications of statistical tools

What role do the various statistical tools reviewed have in the process of investigating anomalous research? The statistical tools allow one to objectively determine how unlikely observations are, and in a sense, a quantitative understanding of how likely data fabrication was. In doing so, the tools provide investigative committees and whistleblowers with more conclusive evidence to ensure that cases of data fabrication are caught and appropriately handled. However, they are not intended to be used as the sole piece of evidence to allege data fabrication; rather, they merely can help to flag studies that should be investigated further. Only a human reviewer—ideally, an expert statistician— could make that determination, usually through a diligent exploration of the study’s results, data, and code.

One could conceive of an algorithm that takes a study’s data and results, runs all the applicable tests (e.g., the NBL, variance analysis, *p* value analysis), and returns informative output. Such information could help editors make informed decisions about accepting papers and help deter data fabrication.

Unfortunately, this application would present serious limitations and practical difficulties. First, by simply applying an algorithm, there is no way to differentiate more suspicious anomalies from those that are more innocuous. An algorithm could not further inspect aspects of a study that seem awry, nor examine studies in their entirety. Additionally, it would be unwise to trust an algorithm alone, since they are only as good as they are designed. Even well-substantiated (and well-meaning) algorithms, such as those for plagiarism, may provide false positive, inflated results. A final decision must be made by a well-informed human. If one wanted to avoid relying on an algorithm, the responsibility of reviewing each study would fall to either journal editors or reviewers. Since both groups tend to have very busy schedules, and proper training to use statistical tools well would take time, it might be more feasible to hire external experts—often called “data sleuths”^12^—with more training to do it instead. It would be challenging to find enough experts who would be willing to review many new articles for one journal, never mind the multitude of existing journals publishing novel psychology research.

Second, “data sleuthing” and alleging fraud also comes with considerable risks. In the past, for example, some accusers of fraud have had their careers ended due to pressures they faced (e.g., see Endnote #1) or have faced legal repercussions from accusers, such as when Francesca Gino sued Data Colada after they reported findings of data falsification to the Harvard Business School (see Endnote #3). Thus, when one suspects fraud based on their own exploration of data or a study, it is essential that one consults with expert statisticians or research integrity officers to verify any evidence. These individuals will be able to confirm whether there exists well-substantiated, thorough evidence to make a claim. Alleging fraud without such evidence is potentially disastrous; as Simonsohn (2013) put it: “Few scholarly goals are as important as eradicating fraud from our journals, and yet few actions are as regrettable as publicly accusing an innocent scholar of fraud” (p. 1886).

Lastly, applying an algorithm also comes with associated disadvantages. One major concern has to do with sharing data. As mentioned, the extent to which papers share data is low, so there has generally been increasing pushes toward encouraging more researchers to partake in data sharing. However, if researchers know that their data might be screened before publication, it may de-incentivize open data sharing due to a perceived hassle or fear of having one’s data falsely flagged. Additionally, as Hartgerink et al. (2016) caution, if data fabricators were particularly insidious, they could utilise the existing tools to ensure their fabricated data sets are undetectable. For example, if they ran the tests and noticed that their reported SDs were abnormally similar, they could simply modify their data set until it “passed” the variance analysis. Thus, implementing tools as a screening measure, while ideal in theory, would be quite challenging to implement in practice.

If not within the realm of screening new research, where else might the tools find use? A more promising use would be to screen previous psychological literature. For example, if there are effects that consistently fail to replicate, it would be worthwhile to investigate the original studies and rule out data fabrication as a potential explanation. Ideally, this would only be done by expert data sleuths with statistical expertise. Beyond screening past research, there would be a benefit to simply raising awareness for readers, journal editors, and reviewers of the “warning signs” of anomalous data or results. Unfortunately, this effort is made more challenging because many anomalies in research studies are emblematic of studies that the current publication incentive structure praises: surprising, headline-worthy, clean results. Since many journals favour such studies, they would be less likely to be detected during the review process. Regardless, making individuals more aware of what data fabrication looks like, and to practise healthy scepticism, would help bring attention toward more instances of potential data fabrication.

## Conclusion

There currently exist several strengths, but many major limitations, for current statistical techniques to detect data anomalies. Moving forward, more research should focus on examining the validity of individual tools to ensure that they are as effective as possible. It is only through ensuring the tools are of high quality that data fraud can be more reliably detected and deterred.

## Supplemental Material

sj-docx-1-tap-10.1177_09593543241311861 – Supplemental material for Tools of the data detective: A review of statistical methods to detect data and result anomalies in psychology Supplemental material, sj-docx-1-tap-10.1177_09593543241311861 for Tools of the data detective: A review of statistical methods to detect data and result anomalies in psychology by Gabriel Crone and Christopher D. Green in Theory & Psychology

## Notes

1. For a striking example of this, see Kate’s story in Chambers (2017, pp. 117–121).

2. The report is no longer available, but see Brown and Heathers (2017a) for a summary.

3. This idea is reinforced by the “Halo” effect: when people are perceived as good, there exists a cognitive bias that makes us believe that said person is more capable, honest, and trustworthy than others (for a review, see Nicolau et al., 2020).

4. Please note that Joenssen (2014) is a preprint and has not been peer reviewed, so may contain errors.

5. Francesca Gino was accused of fabricating data on four of her papers by a blog entitled *Data Colada*, which comprised Uri Simonsohn, Joe Simmons, and Leif Nelson (see Simonsohn et al., 2023a, 2023b, 2023c, 2023d). Primarily, they found anomalies within the data set (e.g., different fonts used, records of rows being moved, abnormal distributions of data, etc.), resulting in Harvard Business School suspending Gino, and Gino retaliating with a lawsuit against *Data Colada* that has since been dropped. For further details on the Francesca Gino case, see Lee (2023).

6. Please note that there is a similar method that aims to investigate the shape of a particular literature’s *p* value distribution called *p* curve analysis (Simonsohn et al., 2014). Because it cannot detect data fabrication, this method should not be confused with *p* value analysis.

7. Please note that this article has not been peer reviewed; it is a preprint and so may contain errors.

8. Please note that Heathers et al.’s (2018) paper is also a preprint and has not been peer reviewed.

9. This value is likely a slight overestimate due to minor errors in the original analysis; see Anaya et al. (2017).

10. Though as will be discussed shortly, this is rarely the case.

11. This is certainly more liberal than the typical .05 level used in psychology!

12. “Data Sleuth” is an informal term used to describe individuals who investigate anomalous research studies. The position itself does not formally exist, though. *Data Colada* (Uri Simonsohn, Joe Simmons, and Leif Nelson), Nick Brown, and James Heathers are all great examples.
