# Supplementary material for: Tools of the data detective: A review of statistical methods to detect data and result anomalies in psychology
Source: Theory Psychol. 2025 Feb 1;35(3):359–80. doi: 10.1177/09593543241311861 (PMC12121900; doi:10.1177/09593543241311861)
Supplement: sj-docx-1-tap-10.1177_09593543241311861 – Supplemental material for Tools of the data detective: A review of statistical methods to detect data and result anomalies in psychology [file sj-docx-1-tap-10.1177_09593543241311861.docx]

**Tools of the data detective supplemental**

The purpose of this supplemental material is to describe the various softwares that exist for the different statistical tools described in the main manuscript.

## *Software for raw data tools*

Since the Newcomb-Benford law (NBL) has been so prevalently studied and applied, there exists many software options in order to conduct one’s own NBL analysis on their data. One such software is an R package called *benford.analysis* (Cinelli, 2018), which has functions to conduct various NBL analyses such as chi-square tests and mean absolute deviation statistics. NBL analyses are also possible to conduct within some popular statistical analysis software such as Jeffreys’s Amazing Statistics Program (JASP; see Ly & Durks, 2021), and Excel (see Collins, 2017).

For multivariate associations, Hartgerink (2024) wrote a package, *ddfab*, that contains a function to perform variance analysis, but in order to run it, one would need to input the means, sample sizes, and standard deviations from comparable literature. Even so, the particular function that produces output merely returns a *p*-value, not the raw test statistic itself. Aside from their software, no designated software exists to automatically perform multivariate analysis. Thus, if one wanted to do a multivariate analysis, they would likely need to do most of the steps manually and, if they have the coding skills, design software to automate particular parts of the process (e.g., code that automatically computed the desired correlation within a given dataset).

## *Software for summary statistics tools*

The only package that exists to perform variance analysis via the Reversed Fisher method is Hartgerink’s (2024) *ddfab* package. The function for the test, however, only returns the *p*-value from the test (and not the bootstrapped distribution). However, performing said bootstrapping would not be too difficult, since there exists other software that are capable of computing bootstrapped estimates (e.g., R’s *boot* package [Canty & Ripley, 2024], SPSS, Stata).

Hartgerink’s (2024) *ddfab* R package also has a function that allows one to run variance analyses (i.e., the *std_var()* function). The function requires manually collecting standard deviations with their accompanying sample sizes within a given article.

Extreme effect sizes lack a designated statistical software. However, software implementations of the technique would be simple, since it would involve running a statistical test, such as a Wilcoxon Test of independence or chi-square test, to see if the target effect size(s) differs from the mean effect size from comparable studies. Such tests can be run in practically any statistical software.

The GRIM and GRIMMER techniques have been implemented within the R package *scrutiny* (Jung, 2024), and simply require a dataset containing the means, standard deviations (SDs), sample sizes, and level of granularity (i.e., what the likert scale ranged from; e.g., if it ranged from 1–7, it would be 7). For SPRITE, Heathers et al. (2018) created three software applications to perform the analyses: a Matlab interface (*mSPRITE*), an R interactive web-app (*rSPRITE*) and a Python-based web app (*pSPRITE*). Recently, Wallrich (2023) reincoprorated the *rSPRITE* app within the R coding environment as a separate package called *rsprite2*.

## *Software Ssummary*

Table 2 summarizes the software that is available for each statistical technique described above.

**Table 2**

*Summary of Existing Software(s) for Each Statistical Technique to Detect Data Fabrication*

| Technique | Software(s) (citation) |
| --- | --- |
| Newcomb-Benford Law | *benford.analysis* R package (Cinelli, 2018)  JASP (see Ly & Durks, 2021)  Excel (see Collins, 2017) |
| Multivariate Associations | *ddfab* R package (Hartgerink, 2024) |
| Variance Analysis | *ddfab* R package (Hartgerink, 2024) |
| Extreme Effect Sizes | No designated software |
| *p*-value Analysis | *ddfab* R package (Hartgerink, 2024) |
| GRIM | *scrutiny* R package (Jung, 2024) |
| GRIMMER | *scrutiny* R package (Jung, 2024) |
| SPRITE | *mSPRITE*, *rSPRITE*, and *pSPRITE* for *Matlab*, R, and Python, respectively (Heathers et al., 2018)  *rsprite2* (Wallrich, 2023) |

#

#

#

#

#

#

#

#

# 
